# Efficacy and safety of a novel topical agent for gallstone dissolution: 2-methoxy-6-methylpyridine

**DOI:** 10.1186/s12967-019-1943-y

**Published:** 2019-06-10

**Authors:** Ho Joong Choi, Suk Joon Cho, Ok-Hee Kim, Jin Sook Song, Ha-Eun Hong, Sang Chul Lee, Kee-Hwan Kim, Sang Kuon Lee, Young Kyoung You, Tae Ho Hong, Eun Young Kim, Jung Hyun Park, Gun Hyung Na, Dong Do You, Jae Hyun Han, Jae Woo Park, Bong Jun Kwak, Tae Yun Lee, Joseph Ahn, Hwan Hee Lee, Seung Kyu Kang, Kyu-Seok Hwang, Jae-Kyung Jung, Kwan-Young Jung, Say-June Kim

**Affiliations:** 1 Department of Surgery, Seoul St. Mary’s Hospital, College of Medicine, The Catholic University of Korea, 222, Banpo-daero, Seocho-gu, Seoul, 06591 Republic of Korea; 2 College of Pharmacy, Chungbuk National University, Cheongju, Republic of Korea; 3 Bio & Drug Discovery Division, Korea Research Institute of Chemical Technology, 141, Gajeong-ro, Yuseong-gu, Daejeon, 34114 Republic of Korea; 4 Catholic Central Laboratory of Surgery, Institute of Biomedical Industry, College of Medicine, The Catholic University of Korea, Seoul, Republic of Korea; 5 Department of Surgery, Daejeon St. Mary’s Hospital, College of Medicine, The Catholic University of Korea, Seoul, Republic of Korea; 6 Department of Surgery, Uijeongbu St. Mary’s Hospital, College of Medicine, The Catholic University of Korea, Seoul, Republic of Korea; 7 Department of Surgery, St. Paul’s Hospital, College of Medicine, The Catholic University of Korea, Seoul, Republic of Korea; 8 Department of Surgery, Bucheon St. Mary’s Hospital, College of Medicine, The Catholic University of Korea, Seoul, Republic of Korea; 9 Department of Surgery, St. Vincent’s Hospital, College of Medicine, The Catholic University of Korea, Seoul, Republic of Korea; 10 Department of Medicinal Chemistry and Pharmacology, University of Science & Technology, Daejeon, Republic of Korea

**Keywords:** 2-Methoxy-6-methylpyridine, Gallstones, Methyl-tert-butyl ether, Topical gallstone-dissolving agent

## Abstract

**Background:**

Although methyl-tertiary butyl ether (MTBE) is the only clinical topical agent for gallstone dissolution, its use is limited by its side effects mostly arising from a relatively low boiling point (55 °C). In this study, we developed the gallstone-dissolving compound containing an aromatic moiety, named 2-methoxy-6-methylpyridine (MMP) with higher boiling point (156 °C), and compared its effectiveness and toxicities with MTBE.

**Methods:**

The dissolubility of MTBE and MMP in vitro was determined by placing human gallstones in glass containers with either solvent and, then, measuring their dry weights. Their dissolubility in vivo was determined by comparing the weights of solvent-treated gallstones and control (dimethyl sulfoxide)-treated gallstones, after directly injecting each solvent into the gallbladder in hamster models with cholesterol and pigmented gallstones.

**Results:**

In the in vitro dissolution test, MMP demonstrated statistically higher dissolubility than did MTBE for cholesterol and pigmented gallstones (88.2% vs. 65.7%, 50.8% vs. 29.0%, respectively; *P* < 0.05). In the in vivo experiments, MMP exhibited 59.0% and 54.3% dissolubility for cholesterol and pigmented gallstones, respectively, which were significantly higher than those of MTBE (50.0% and 32.0%, respectively; *P* < 0.05). The immunohistochemical stains of gallbladder specimens obtained from the MMP-treated hamsters demonstrated that MMP did not significantly increase the expression of cleaved caspase 9 or significantly decrease the expression of proliferation cell nuclear antigen.

**Conclusions:**

This study demonstrated that MMP has better potential than does MTBE in dissolving gallstones, especially pigmented gallstones, while resulting in lesser toxicities.

**Electronic supplementary material:**

The online version of this article (10.1186/s12967-019-1943-y) contains supplementary material, which is available to authorized users.

## Background

Cholelithiasis is a highly prevalent disease, particularly in developed countries, with incidence rates of 10–15% in the adult population, indicating that 20–25 million Americans have gallstones [1]. Laparoscopic cholecystectomy is considered the cornerstone treatment for symptomatic cholelithiasis. However, clinicians are occasionally confronted with inoperable patients because of the operative risks or refusal of patients. To better cope with such situations, several topical gallstone-dissolving agents have been developed, which are administrated via the percutaneous transhepatic route.

Of all the gallstone-dissolving compounds, methyl tert-butyl ether (MTBE) is a representative solvent and the only such drug currently used. MTBE is an alkyl ether similar to diethyl ether, an anesthetic agent. Unlike the lower boiling point of diethyl ether (35 °C), MTBE has a relatively higher boiling point (55 °C); thus, it is liquid at body temperature [2, 3]. Acceptable dissolubility of MTBE was validated by a variety of studies, including a large survey of 803 patients across 21 European hospitals that demonstrated a dissolution rate of 96.6% (724/804) with a low rate of toxic side effects and complications [4]. However, the widespread use of MTBE is limited by its side effects, such as nausea, upper abdominal pain, duodenitis, mild-to-moderate anesthesia, and hemolysis [5–14]. A substantial proportion of these side effects could be attributed to its relatively low boiling point and the resultant higher evaporation; the evaporation rate of MTBE is 8.0 (i.e., 8.0 times higher than that of standard *n*-butyl acetate), and thus is classified as “fast evaporating”.

In this study, we first developed a gallstone-dissolving compound with an aromatic moiety, named 2-methoxy-6-methylpyridine (MMP). To date, various compounds have been used to dissolve gallstones, including MTBE, heparin, bile acid, d-limonene, mono-octanoin, ethylenediamine tetraacetic acid (EDTA), and sodium hexametaphosphate, all of which are composed of aliphatic chains [15, 16]. MTBE has an asymmetric chemical structure that contains an ether functional group. With an oxygen atom in between, one side of MTBE is the bulky tert-butyl group and the other side is a simple methyl group. In MMP, the bulky aliphatic tert-butyl group of MTBE is replaced with pyridine, an aromatic group. The compounds with an aromatic ring tend to have a relatively higher boiling point and lower vapor pressure [17]. MMP, thus, has a relatively higher boiling point (156 °C) and evaporates less, raising the possibility of having lesser toxicities while maintaining similar dissolubility as MTBE. In this study, we intended to determine whether MMP could be an alternative to MTBE by comparing their gallstone dissolubility and toxicities.

## Methods

### Chemicals and reagents

MTBE was obtained from Sigma-Aldrich (St. Louis, MO). MMP was produced in the Korea Research Institute of Chemical Technology (KRICT, Daejeon, Republic of Korea).

### Nuclear magnetic resonance spectroscopy

Nuclear magnetic resonance (NMR) was used to measure the chelation effect of the gallstone-dissolving compounds, MTBE and MMP. 1-h NMR spectra were recorded on Bruker INOVA 400 MHz NMR spectrometers (BRUKER, Fȁllanden, Switzerland) at 25 °C. Chemical shifts are reported in parts per million (ppm). Data for 1-h-NMR are reported as follows: chemical shift (δ ppm) (integration, multiplicity, and coupling constant [Hz]). Multiplicities are reported as follows: s = singlet, d = doublet, t = triplet, q = quartet, sep = septet, dd = doublet of doublets, and m = multiplet. The residual solvent peak was used as an internal reference.

### Thermogravimetric analysis

The thermogravimetric analysis (TGA), differential thermal analysis (DTA), and derivative of TGA (DTG) curves were recorded for each solvent using Thermo plus EVO II TG 8120 series thermal analyzer (RIGAKU, Tokyo, Japan). All three thermo-curves, TG, DTG, and DTA, were recorded simultaneously within temperatures of 25–100 °C in an air atmosphere. The thermo-curves were recorded for heating rates of 5 °C/min. In all the thermo-curves, the sample mass initially weighed between 4 and 5 mg. The kinetic parameters were calculated using the data obtained from the recorded thermo-curves by employing non-mechanistic Kissinger evaluation method.

### Determination of gallstone components

We used the following assay kits to determine the components of gallstones; Cholesterol Enzymatic Assay Kit (Xpressbio, Fredrick, MD) for cholesterol, Triglyceride Quantification Kit (Cell Biolabs, San Diego, CA) for triglycerides, Bilirubin Assay Kit (Cell Biolabs) for bilirubin, and Phosphate Colorimetric Assay Kit (Biovision, Mountain View, CA) for bilirubin. The gallstones were homogenized with the substances described in each assay kit and the supernatant was collected for assay. After incubation for a determined period at 37 °C in the assay buffer, the absorbance was measured using a microplate reader (model 680; Bio-Rad, Hercules, CA).

### Measurement of in vitro gallstone dissolubility

We collected gallstones following cholecystectomy in patients with gallstones. The study was approved by the Ethics Committee of Daejeon St Mary’s hospital, the Catholic University of Korea (IRB code: DC09FZZZ0045). We first determined the cholesterol contents in the collected gallstones using Cholesterol Enzymatic Assay Kit (Xpressbio, Fredrick, MD). Based on the cholesterol content, the gallstones were categorized into cholesterol (cholesterol content > 70%), mixed (70–30%), and pigmented gallstones (< 30%). The gallstones were air-dried, weighed, and preserved in saline. Subsequently, they were matched for size, weight, and shape for preparation for the dissolubility measurements. The three types of gallstones, with a mean weight of 43 ± 4 mg, were placed in separate glass containers, to which 10 ml aliquots of MTBE or MMP were added; the aliquots were aspirated and replaced every hour. The glass containers were gently stirred at 50 rpm on the reactor (VS-8480SF; Vision Co., Daejeon, Republic of Korea) at 37 °C for 24 h. Gallstone dissolubility according to each solvent was determined by measuring the dry weights of the gallstones at three different intervals (4, 8, and 24 h). Specifically, in vitro gallstone dissolubility (%) of each solvent was defined as (the average weight of untreated gallstones − the average weight of gallstones after solvent treatment)/the average weights of untreated gallstones.

### Cell viability assay

Cell viability of human gallbladder epithelial cells (hGBECs) was evaluated with 2-(4-iodophenyl)-3-(4-nitrophenyl)-5-(2,4-disulfophenyl)-2H-tetrazolium (water soluble tetrazolium salt; ST-1) assay using EZ-Cytox Cell Proliferation Assay kit (Itsbio, Seoul, Republic of Korea) according to the manufacturer’s instructions. Additionally, we performed another series of cell viability assay for the following cells using Cyto X cell viability assay kit (LPS solution Co., Daejeon, Republic of Korea); Vero (African green monkey kidney, ATCC#81-CCL), L929 (mouse fibroblast, ATCC #2148-CRL), NIH 3T3 (mouse embryonic fibroblast, ATCC #1658-CRL), and CHO-K1 (Chinese hamster ovary, ATCC #61-CCL) cells.

### In vivo animal experiments

#### In vivo dissolubility study using a hamster model of gallstones

All animal studies were carried out in compliance with the guidelines of the Institute for Laboratory Animal Research in Korea. This animal study was approved by the Institutional Animal Care and Use Committee of the Clinical Research Institute at Daejeon St. Mary’s Hospital at the Catholic University of Korea (IRB No. CMCDJ-AP-2016-004). For in vivo validation of each solvent, we used 10-week-old female Syrian golden hamsters (*Mesocricetus auratus*, Harlan Sprague–Dawley Indianapolis, IN). Hamsters in each group were fed a different diet as follows for 4 months: the hamsters in the control group (N = 10) were fed a normal diet, while those in group CG (the group with cholesterol gallstones, N = 50) were fed a general rodent diet with 0.5% cholesterol, and those in group PG (the group with pigmented gallstones, N = 50) were fed a diet rich in carbohydrates. The diet compositions of each group are shown in Additional file 1: Table S1. The body weights of the hamsters were measured weekly. After 4 months of diet, the hamsters with gallstones were identified using abdominal ultrasonography and selected for further experiments. We directly infused the control material (DMSO) and each solvent into the gallbladder of the hamsters with cholesterol (n = 40) and pigmented gallstones (n = 40), respectively, after laparotomy under general anesthesia. After laparotomy under general anesthesia, each solvent was infused into the gallbladder using a 30-gauge syringe; after complete aspiration of the bile, the gallbladder was cautiously filled with a similar volume (0.1 ml) of dimethyl sulfoxide (DMSO), MTBE, and MMP, respectively. After 24 h, the hamsters were euthanized, and the gallstones and several pieces of tissues (liver, kidney, and gallbladder) were collected for further evaluations. Gallstone dissolubility of each solvent was determined by comparing the weights of solvent-treated gallstones and control (DMSO)-treated gallstones. Specifically, in vivo gallstone dissolubility (%) of each solvent was defined as (the average weight of DMSO-treated gallstones − the average weight of gallstones after solvent treatment)/the average weights of DMSO-treated gallstones.

#### In vivo acute toxicity test in mice

Seven-week-old female Institute of Cancer Research (ICR) mice were purchased from Orient Bio, Inc. (Seongnam, Republic of Korea). The mice were allowed to acclimatize to their new environment for 7 days and were maintained in an environment with temperature 23 ± 3 °C, humidity 50 ± 10%, 12-h light–dark cycle with 150–300 lx, and ventilation at 10–20 times/h. For the acute toxicity study, the mice were divided into 3 groups (n = 7, respectively). After starvation for 24 h, the control group was given 5% DMSO, while the treatment groups received MTBE and MMP, respectively, at 2000 mg/kg prepared in a corn oil through oral gavage. The general behavior (skin, respiration, tremors, lethargy, or sleep), body weight, and mortality were monitored for 14 days.

#### Measurement of Zebrafish locomotor activity according to each solvent

Zebrafish were maintained under standard conditions as previously reported [18–20]. To monitor the locomotor activity, larval zebrafish at 5 days post-fertilization were placed in individual wells of a 96-well plate with 100 μl embryonic medium. Then, 100 μl of MTBE and MMP were added to a final concentration of 1 mM, respectively. After the treatment, changes were monitored at 28 °C for 60 min using DanioVision and EthoVision 10 XT locomotion tracking software (Noldus, Netherlands).

### Statistical analysis

All data were analyzed with SPSS 11.0 software (SPSS Inc., Chicago, IL), and are presented as mean ± standard deviation (SD). Statistical comparisons between the groups were determined using the Kruskal–Wallis test. P < 0.05 was considered statistically significant. All authors had access to the study and reviewed and approved the final manuscript.

### Additional materials and methods

Additional and more detailed information regarding the experimental procedures are fully described in Additional file 2: Materials and methods.

## Results

### Determination of basic characteristics of MMP

**Fig. 1:**
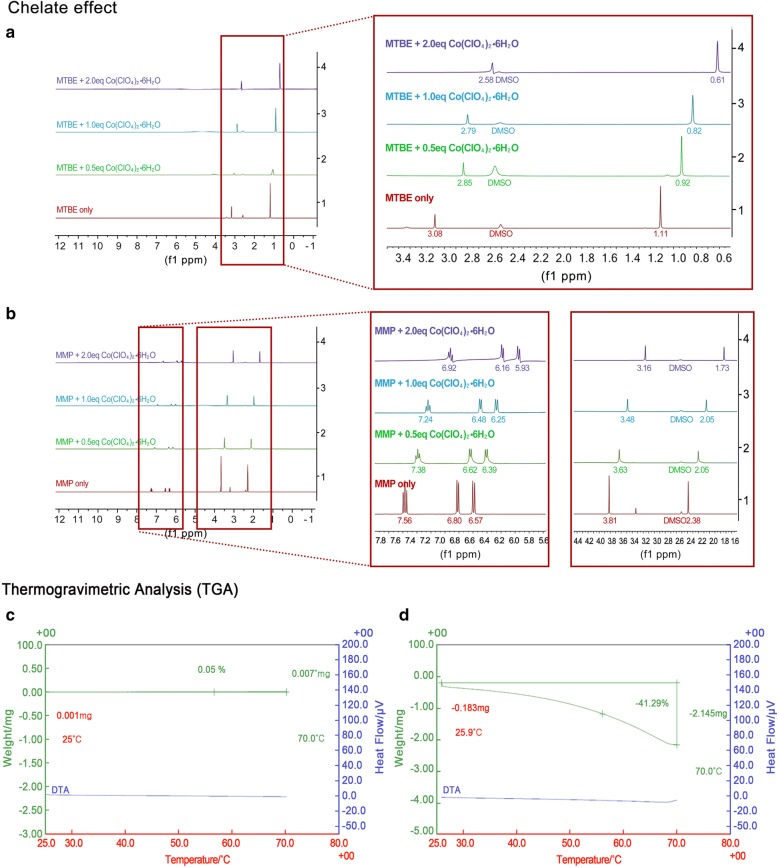
Validation of the chelate effect and thermogravimetric analysis of each solvent. **a** Determination of intrinsic proton chemical shifts of MTBE in the presence of Co^2+^ cation using proton nuclear magnetic resonance (NMR). Net chemical shift values (Δδ) of MTBE with increasing Co^2+^ cation concentrations were 0.19 (0–0.5 eq. Co^2+^), 0.10 (0.5–1.0 eq. Co^2+^), and 0.21 (1.0–2.0 eq. Co^2+^). **b** Determination of intrinsic proton chemical shifts of MMP in the presence of Co^2+^ cation using proton nuclear magnetic resonance (NMR). Net chemical shift values (Δδ) of MMP with increasing Co^2+^ cation concentrations were 0.18 (0–0.5 eq. Co^2+^), 0.14 (0.5–1.0 eq. Co^2+^), and 0.32 (1.0–2.0 eq. Co^2+^). There was no difference in the chemical shifts between MTBE and MMP in the presence of 0.5 equivalent Co^2+^. However, the chemical shift of MMP was more prominently increased, possibly due to the chelation strength between MMP and Co^2+^. **c** Thermogravimetric analysis of MTBE. TGA can be used to evaluate the thermal stability of a compound. A thermogravimetric analyzer continuously measures the mass of a substance (MTBE or MMP) while the temperature is changed over time. In the case of MTBE, TGA analysis was impossible because it quickly vaporized in the chamber due to the low boiling point. **d** Thermogravimetric analysis of MMP. In the case of MMP, it did not vaporize before applying the initial heat due to its higher boiling point of 156 °C, and slowly vaporized as the temperature increased. MMP, 2-methoxy-6-methylpyridine; MTBE, methyl tert-butyl ether; NMR, nuclear magnetic resonance; TGA, thermogravimetric analysis

Table 1 summarizes the structural and functional comparisons between MTBE and MMP. MMP has a structure wherein the tert-butyl group in MTBE is replaced by a bulky aromatic functional group, thus, resulting in elevation of the boiling point from 55 (MTBE) to 156 °C (MMP). A substantial proportion of MTBE toxicities are related to higher evaporation caused by a relatively low boiling point; therefore, MMP is expected to have relatively lesser toxicity due to its higher boiling point. Additionally, contrasted by MTBE, which has only one heteroatom oxygen, MMP has two heteroatoms (nitrogen and oxygen) in the form of a tweezer, which increases the possibility of stably trapping the cations in gallstones by chelate effect, which refers to an enhanced affinity toward a metal atom by a compound that is attached to the metal atom in a cyclic or ring structure. Chelate effect accelerates the dissolution of gallstones, as demonstrated by EDTA, which potentiates calcium stone dissolution by chelating the calcium component of gallstones [21]. We herein attempted to determine the strength of chelate effect of each solvent (MTBE and MMP) using proton NMR, because the chemical shift of the intrinsic proton is expected to change according to the chelate effect between lone-pair electrons of the heteroatom and cation (Fig. 1a, b). To validate this concept, we used Co(ClO_4_)_2_·6H_2_O as the Co^2+^ provider because Co(ClO_4_)_2_·6H_2_O is likely to have independent cations due to the bulky anion ClO_4_^2−^. When 0.5 equivalent of Co(ClO_4_)_2_·6H_2_O was added to solvents (MTBE and MMP), the chemical shifts were negligible (0.19 and 0.18 ppm, respectively). However, when the equivalent of Co(ClO_4_)_2_·6H_2_O was increased to 1.0–2.0 relative to the solvents, MMP exhibited considerably higher chemical shift than did MTBE (0.33–0.65 ppm vs. 0.50–1.03 ppm) (Fig. 1a, b), suggesting an enhanced affinity by chelate effect between MMP and Co^2+^. Other data supporting enhanced chelate effect by MMP were presented in Additional file 3: Fig. S1, Additional file 4: Fig. S2, Additional file 5: Fig. S3, Additional file 6: Fig. S4, Additional file 7: Fig. S5, and Additional file 8: Fig. S6.

**Table 1:** Comparison between MTBE (methyl tertiary-butyl ether) and MMP (2-methoxy-6-methylpyridine)

	MTBE	MMP
Structure		
Molecular weight (g/mol)	88.15	123.26
Boiling point (°C)	55	156
Property	High vaporization	Low vaporization
Feature	Bulky aliphatic chain ① Bulky aliphatic chain ② Hydrophobic	Bulky heterocyclic ring ① Bulky aromatic ring ② Heteroatom ③ Hydrophobic and hydrophilic ④ Chelation effect
Toxicity	Many toxicities have been reported	Less toxicity compare with MTBE

Next, TGA was used to determine the stability of each solvent with increasing temperatures (Fig. 1c, d). In MTBE, we could not attain TGA data, because MTBE was already volatile at 25 °C and left no analytical residue. However, MMP progressively evaporated as the temperature increased from 20 to 70 °C and demonstrated the most abrupt change at 57 °C.

### Classification of gallstones according to their components

**Fig. 2:**
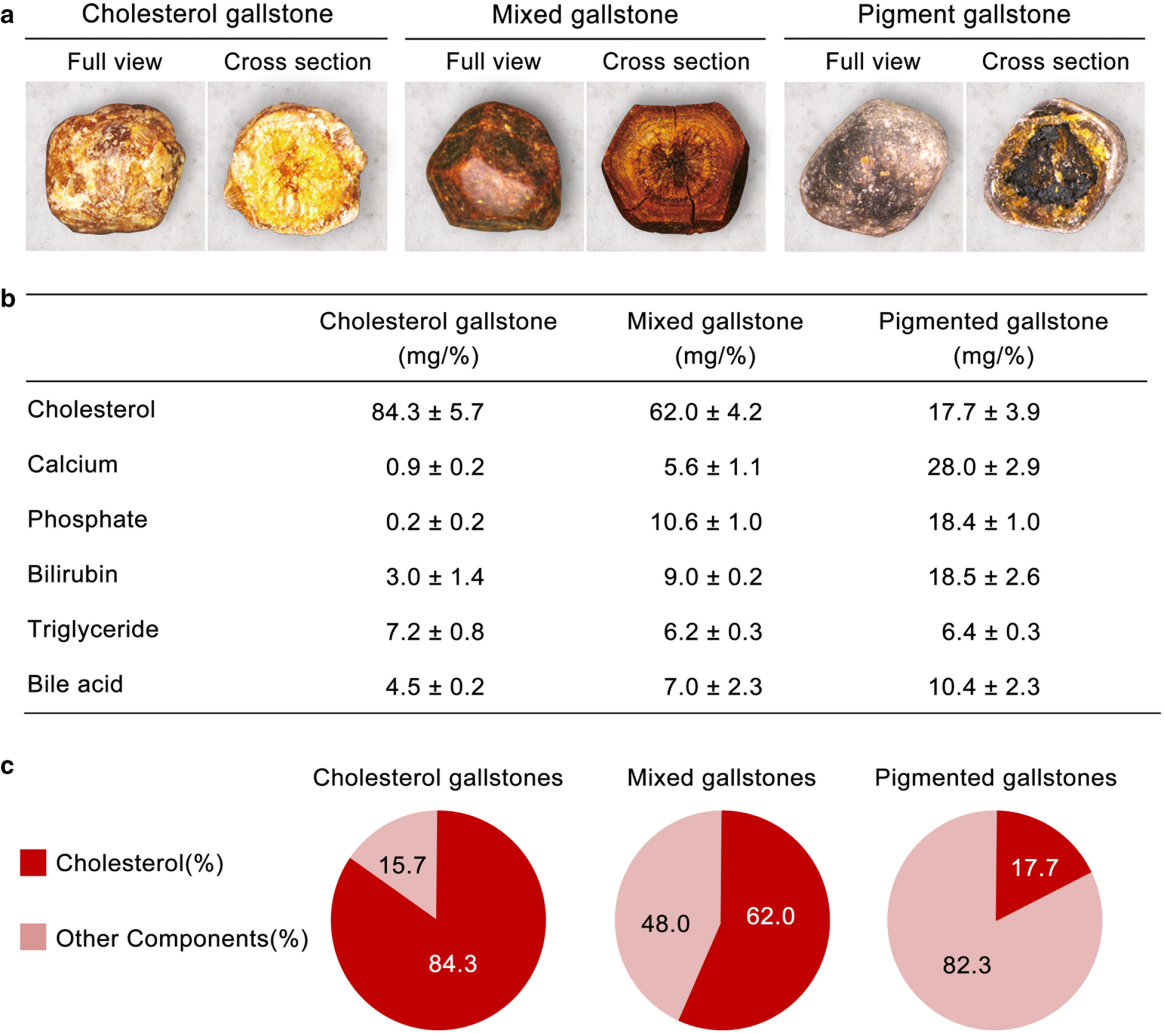
Compositions of gallstones according to cholesterol content. **a** Representative gross and cross-sectional appearances of cholesterol, mixed, and pigmented gallstones. **b** Comparison of the components of gallstones according to their subtypes (cholesterol, mixed, and pigmented gallstones). **c** The percent (%) of cholesterol relative to the sum of other components in each type

Based on the literature, we classified gallstones based on their cholesterol content (≥ 70%, 70–30%, and < 30%) into cholesterol, mixed, and pigmented gallstones, respectively [22]. Figure 2a shows the representative gross and cross sectional appearances of each type of gallstones. Cholesterol enzymatic assay revealed that the cholesterol contents were 84.3 ± 5.7% for cholesterol stones, 62.0 ± 3.9% for mixed stones, and 17.7 ± 0.2% for pigmented stones (Fig. 2b, c). In addition to cholesterol content, gallstones of each type demonstrated quite different compositions from other types of gallstones; for instance, pigmented stones contained significantly higher concentrations of phosphate and calcium than cholesterol stones.

### Effects of each solvent on human gallbladder epithelial cells

**Fig. 3:**
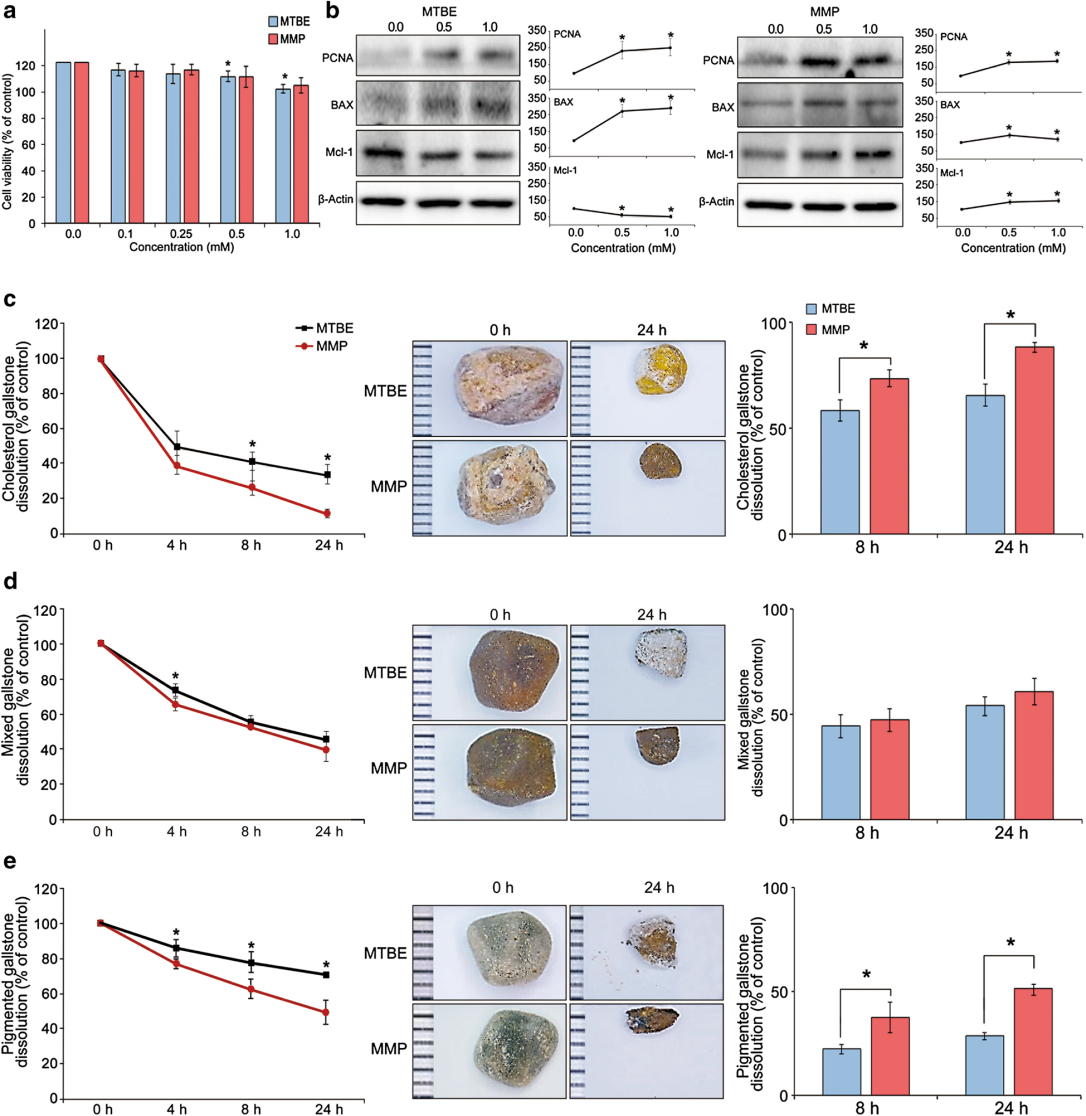
In vitro determination of toxicities and gallstone dissolubility of each solvent. **a** Cell viability assay showing the effects of each solvent on the viability of human gallbladder epithelial cells (hGBECs). The viability of GBECs did not considerably change according to the concentration of MTBE or MMP (0.0–1.0 mM) over 24 h. **b** Western blot analysis showing the effects of MTBE and MMP on the expression of PCNA (proliferation marker), Mcl-1 (anti-apoptosis marker), and BAX (apoptosis marker). [Top] With rising MTBE concentrations, there were sharp rises in the expression of PCNA and BAX, and fall in the expression of Mcl-1. [Bottom] With rising MPP concentrations, the expression of PCNA was less prominently increased than that of MTBE. In addition, unlike to MTBE, the expression of BAX was decreased at higher concentration (1 mM), and the expression of Mcl-1 was increased. **c** Dissolubility of cholesterol gallstones. [Left] Time-response graph of cholesterol gallstones. MMP dissolved the cholesterol gallstones significantly better than MTBE after 8 h and 24 h (*P* < 0.05). [Middle] Representative pictures of the residual gallstones at 24 h. [Right] Comparison of dissolubility of each solvent after 8 h and 24 h in cholesterol gallstones. **d** Dissolubility of mixed gallstones. [Left] Time-response graph of mixed gallstones. The two solvents demonstrated similar dissolutions in mixed gallstones, except for a higher temporal dissolution of MMP at 4 h. [Middle] Representative pictures of the residual gallstones after 24 h. [Right] Comparison of dissolubility of each solvent after 8 h and 24 h in mixed gallstones. **e** Dissolubility of pigmented gallstones. [Left] Time-response graph of pigmented gallstones. MMP dissolved the pigmented gallstones significantly better than MTBE after 4 h, 8 h, and 24 h (*P* < 0.05). [Middle] Representative pictures of the residual gallstones after 24 h. [Right] Comparison of dissolubility of each solvent after 8 h and 24 h in pigmented gallstones. Values are presented as mean ± standard deviation of three independent experiments. The in vitro gallstone dissolubility (%) of each solvent was defined as (the average weight of untreated gallstones − the average weight of gallstones after solvent treatment at specific time interval)/the average weights of untreated gallstones. *P < 0.05. BAX, Bcl-2-like protein 4; Mcl-1, myeloid cell leukemia 1; MMP, 2-methoxy-6-methylpyridine; MTBE, methyl tert-butyl ether; PCNA, proliferation cell nuclear antigen

We investigated the direct toxicities of each solvent on hGBECs. Cell viability tests revealed that the viability of hGBECs did not significantly decrease with elevating concentrations of MTBE and MMP up to 1.0 M, suggesting the negligible effects of both solvents on the viability of GBECs (Fig. 3a). Next, we performed Western blot analysis to determine the expression of the markers that reflect cell proliferation (PCNA), anti-apoptosis (Mcl-1), and apoptosis (BAX) with increasing concentration of each solvent (Fig. 3b). With increasing MTBE concentrations, there were sharp rises in the expression of PCNA and BAX, and fall in the expression of Mcl-1. MMP induced similar changes in these markers as MTBE; however, the changes were not as prominent as MTBE, and there was even a drop in the expression of BAX at a higher concentration (1 mM).

### In vitro determination of gallstone dissolubility by each solvent

To determine the dissolubility of each solvent according to the type of gallstone, we compared the dry weights of human gallstones at three different time-points (4, 8, and 24 h) after they directly reacted with each solvent. Gallstone dissolubility was calculated as the difference in the weights of gallstones between pretreatment and the determined time intervals. In cholesterol gallstones, MMP dissolubility was significantly better than MTBE dissolubility at 8 h and 24 h (*P* < 0.05) (Fig. 3c). In mixed gallstones, the two solvents demonstrated similar dissolubility, except for temporal higher dissolubility of MMP at 4 h (Fig. 3d). In pigmented gallstones, MMP dissolubility was significantly better than MTBE dissolubility at 4 h, 8 h and 24 h (*P* < 0.05) (Fig. 3e). After 8 h, whereas MTBE demonstrated 58.5%, 44.5%, and 22.2% dissolubility, MMP demonstrated 73.5%, 47.5%, and 37.2% dissolubility for cholesterol, mixed, and pigmented gallstones, respectively. Furthermore, after 24 h, whereas MTBE showed 65.7%, 54.2%, and 29.0% dissolubility, MMP showed 88.2%, 61.0%, and 50.8% dissolubility for cholesterol, mixed, and pigmented gallstones, respectively.

### Determination of systemic effects and in vivo gallstone dissolubility by each solvent

We established the hamster models having cholesterol gallstones (CG group) and pigmented gallstones (PG group) with the individual 3-month diet protocols as described earlier [23]. During the course of feeding, the hamsters of each group showed consistent increases in body weights (Additional file 9: Fig. S7), and at the end of the feeding period, gallstones were found in 88% (44/50) of CG group, and 82% (41/50) of PG group. After detecting gallstones by abdominal ultrasonography, we randomly assigned the hamsters with cholesterol gallstones (N = 40) and pigmented gallstones (N = 40) into DMSO (control; n = 10), MTBE (n = 15), and MMP (n = 15) groups, respectively. Subsequently, we directly infused each material into the gallbladder of the hamsters with each type of gallstones, respectively, after laparotomy under general anesthesia.

**Fig. 4:**
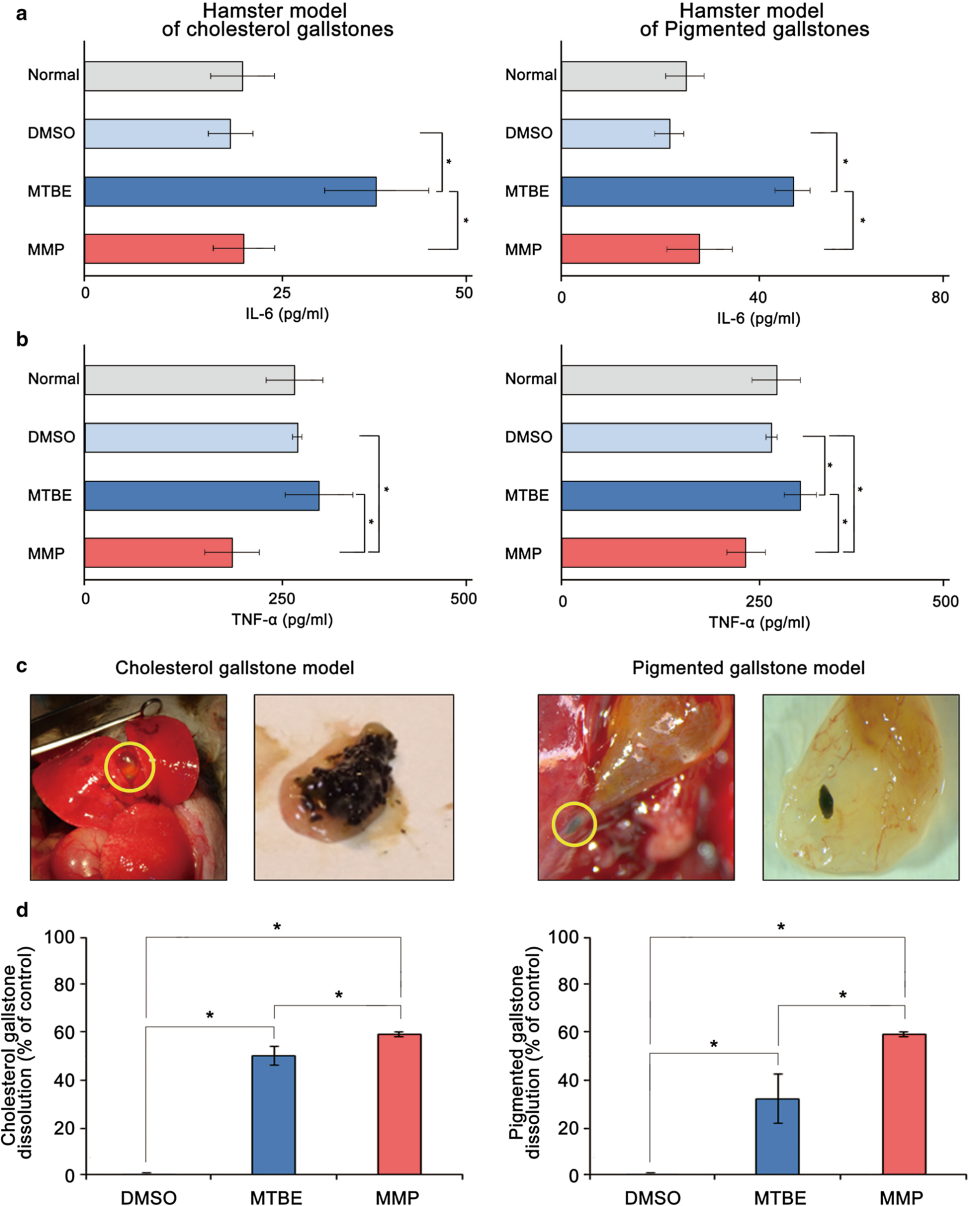
Validation of the effects of each gallstone-dissolving compound in the hamster model of gallstones. **a** Effects of intracystic infusion of each solvent on the release of pro-inflammatory mediators in hamsters with gallstones. ELISA shows that while MTBE significantly increased the levels of IL-6, MMP did not in both hamster models of cholesterol [Left] and pigmented gallstones [Right]. **b** Effects of intracystic injection of each solvent on the release of TNF-α in hamsters with gallstones. In contrast with MTBE, MMP decreased the serum levels of TNF-α in both hamster models of cholesterol [Left] and pigmented gallstones [Right]. **c** Representative pictures demonstrating the development of cholesterol [Left] and pigmented [Right] gallstones in the experimental hamsters following their respective protocol diets. Yellow circles indicate the gallbladder and gallstones within the gallbladder. **d** Demonstration of in vivo gallstone dissolubility of each solvent. After infusing each solvent into the gallbladders of hamsters with gallstones for 24 h, gallstone dissolubility was determined by comparing the weights of the residual gallstones. The in vivo gallstone dissolubility (%) of each solvent was defined as (the average weight of DMSO-treated gallstones − the average weight of gallstones after solvent treatment)/the average weights of DMSO-treated gallstones. MMP demonstrated significantly higher dissolubility than MTBE in both hamster models of cholesterol [Left] and, especially, pigmented [Right] gallstones. The dissolubility of each solvent (MTBE and MMP) for pigmented gallstones was 32.0% and 54.3%, respectively (P < 0.05). *P < 0.05. DMSO, dimethyl sulfoxide; MMP, 2-methoxy-6-methylpyridine; MTBE, methyl tert-butyl ether

First, to determine whether solvent infusion evokes a systemic inflammatory response, we measured the serum concentrations of proinflammatory mediators (IL-6 and TNF-α) by ELISA at 24 h of intracystic infusion. While MTBE significantly increased the serum levels of IL-6, MMP did not in both hamster models of cholesterol and pigmented gallstones (Fig. 4a). Additionally, whereas MTBE increased the serum levels of TNF-α, MMP decreased the levels of TNF-α in both models (Fig. 4b). Subsequently, we determined the dissolubility of each solvent by comparing the weights of the residual gallstones at 24 h after infusion (Fig. 4c). DMSO was used as the control material. In CG group, MTBE demonstrated 50.0% dissolubility, while MMP demonstrated 59.0% dissolubility, and the difference was statistically significant (*P *< 0.05) (Fig. 4d, left). Moreover, PG group was found to have far more significant differences; whereas MTBE showed 32.0% dissolubility, MMP showed 54.3% dissolubility (*P *< 0.05) (Fig. 4d, right). Therefore, we could conclude that MMP exhibited significantly higher dissolubility than MTBE in the hamster models with gallstones, especially pigmented gallstones.

### Determination of in vivo toxicity of each solvent

**Fig. 5:**
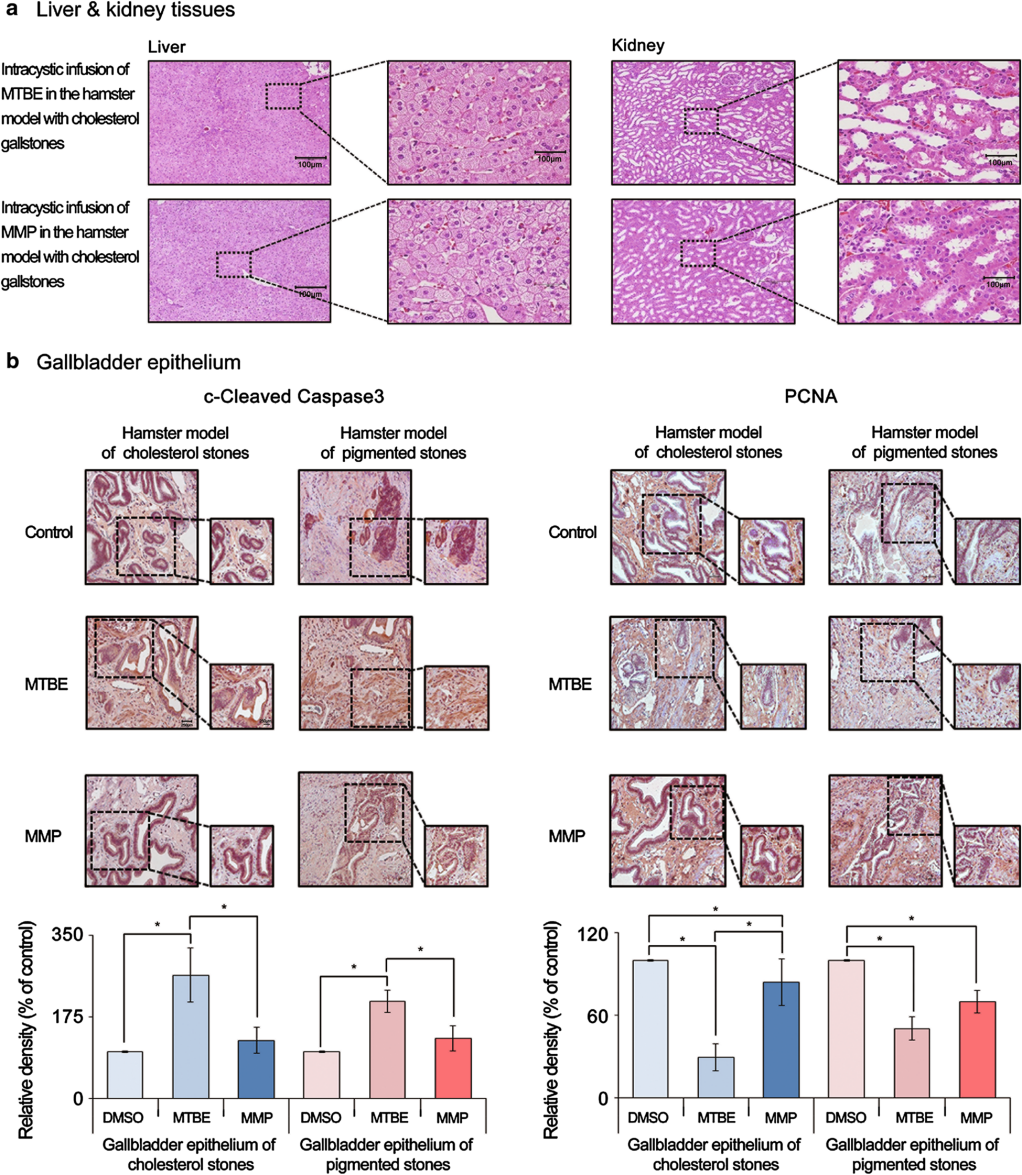
Validation of tissue toxicities of each gallstone-dissolving compound in hamster models of gallstones. **a** Hematoxylin–Eosin (H&E) stains of the liver and kidney tissues obtained from the gallstone-containing hamsters at 24 h after intracystic injection of each solvent. In HE stains, the liver and kidney tissues of each group appeared to be well-preserved and did not demonstrate any signs of injury. **b** [Left] Cleaved caspase-3 immunohistochemical stains of the gallbladder tissues obtained from the hamsters with gallstones at 24 h after intracystic injection of each solvent. Whereas MTBE significantly increased the expression of cleaved caspase-9 (*P* < 0.05), MMP did not in both hamster models of cholesterol and pigmented gallstones. [Right] PCNA immunohistochemical stains of the gallbladder tissues obtained from the hamsters with gallstones at 24 h after intracystic injection of each solvent. Although both solvents significantly decreased the expression of PCNA, the reduction in PCNA expression was far significant after MTBE than it was after MMP in both gallstone models (*P* < 0.05). *P < 0.05. DMSO, dimethyl sulfoxide; MMP, 2-methoxy-6-methylpyridine; MTBE, methyl tert-butyl ether

To determine the toxic effects of each solvent on the liver and kidney, we performed histological evaluations using the tissues obtained from the hamsters at 24 h after intra-cystic infusion of each solvent. On microscopic observation with hematoxylin and eosin stains, the liver and kidney tissues of both solvent-treated hamsters were well preserved and did not show any visible signs of injury (Fig. 5a). Next, to determine the direct tissue toxicity of each solvent, we performed immunohistochemical (IHC) analysis of the gallbladder specimens obtained from the solvent-treated hamsters. The IHC panel included stains for representative markers of pro-apoptosis (cleaved caspase-3) and proliferation (PCNA). The expression of cleaved-caspase 3 was far more prominent after MTBE infusion than it was after MMP infusion in both CG and PG groups (*P* < 0.05) (Fig. 5b, left). Additionally, MTBE infusion far more significantly decreased the expression of PCNA than did MMP infusion (*P* < 0.05), suggesting a higher toxicity of MTBE over MMP, particularly in CG group (Fig. 5b, right).

### Comprehensive testing of in vitro and in vivo toxicities of each solvent in various models

**Fig. 6:**
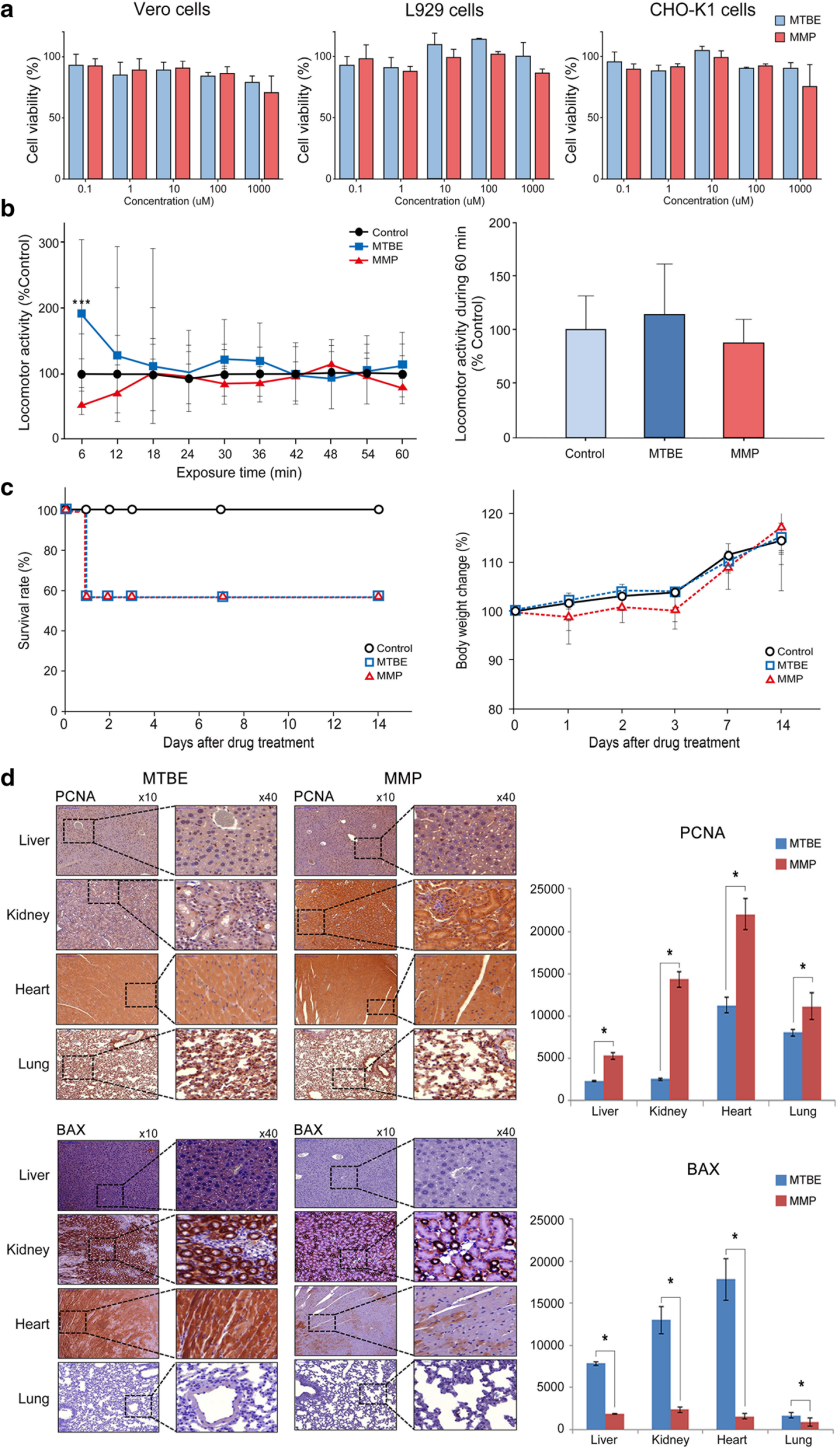
Validation of toxicities of each gallstone-dissolving compound in various in vitro and in vivo models. **a** In vitro cytotoxicity of each solvent in Vero, L929, and CHO-K1 cells. To compare the in vitro toxicities of MTBE and MMP, cell viability assay was performed using Cyto X cell viability assay kit. The viability of Vero, L929, and CHO-K1 cells did not considerably change according to the concentration of MTBE or MMP (0–1000 μM) over 24 h. **b** [Right] The effect of each solvent on locomotor activity in larval zebrafish. For determining the CNS toxicity of each solvent, we tracked larval zebrafish locomotion after 1 mM MTBE and 1 mM MMP treatment, respectively, over 60 min. The locomotor activity was normalized against DMSO control and presented as a percentage. While MTBE increased the locomotor activity during the first 6 min after treatment, MMP reduced it during the first 6 min after treatment. However, overall, each group did not show any significant difference in the total locomotor activity when compared with DMSO control. [Left] Total locomotor activity during 60 min, demonstrating no significant difference between MTBE and MMP groups. **c** Survival rate and body weight changes after oral administration of MTBE and MMP in mice. [Left] Compared to the control group, MTBE and MMP groups demonstrated similar survival patterns. [Right] MMP group demonstrated slight reduction in body weight during 7 days, which recovered by 14 days after the treatment. **d** Determination of tissue toxicities of each solvent on 14 days after oral administration. [Top] PCNA immunohistochemical stains. The mice treated with MMP showed higher expression of PCNA (a proliferation marker) than the mice treated with MTBE (*P* < 0.05). [Bottom] BAX immunohistochemical stains. The mice treated with MMP showed lesser expression of BAX (an apoptotic marker) than the mice treated with MTBE (*P* < 0.05). *P < 0.05. BAX, bcl-2-like protein 4; MMP, 2-methoxy-6-methylpyridine; MTBE, methyl tert-butyl ether; PCNA, proliferation cell nuclear antigen

To further determine the in vitro toxicities of each solvent, we performed cell viability assay of various cell lines, including Vero cells, L929 cells, and CHO-K1 cells (Fig. 6a). We found that both solvents did not decrease cell viability of these cell lines, at least up to 1.0 M concentration of each solvent.

Next, for determining the central nervous system (CNS) toxicity of each solvent, we tracked larval zebrafish locomotion in 1 mM MTBE and MMP, respectively, over 60 min (Fig. 6b). The locomotor activity was normalized against DMSO as the control, and presented as a percentage. It was found that MTBE increased the locomotor activity during the first 6 min; however, MMP reduced it during the same period. Consequently, such differences were offset when the total sum of locomotor activities were counted over the observation period.

Finally, to determine the in vivo acute toxicity arising from a single exposure, the mortality, body weight changes, and general behaviors of the mice were monitored during 14 days after oral administration of each solvent at a dose of 2000 mg/kg (Fig. 6c). On the second day of the experiment, the same number of mice (3/7, 42.9%) were found to be dead in both groups administrated either MTBE or MMP, respectively. Those animals presented abnormal behaviors, including wheezing, tremors, and ataxia until death without noticeable differences between each group. Similar, but not pronounced, behaviors were also observed in the survived animals of the same group, and the behavior patterns were similar in both groups. In addition, the mice administrated with MMP were found slightly underweight during the first 7 days and, subsequently, recovered thereafter. Subsequently, after euthanizing the mice on 1 day and 14 days after oral administration, respectively, we obtained the specimens of liver, kidney, heart and lung, for the determination of histological effects of each solvent. Immunohistochemistry was performed for the markers of proliferation (PCNA), apoptosis (caspase 3 and Bax), and anti-apoptosis (survivin). On 1 day (Additional file 10: Fig. S8) as well as 14 day, the mice treated with MMP showed higher expression of PCNA, lesser expression of caspase 3 and Bas, and higher expression of survivin than the mice with MTBE (all *P*s < 0.05) (Fig. 6d and Additional file 11: Fig. S9), suggesting the lesser tissue toxicity of MMP.

## Discussion

This is the first validation report of MMP, the gallstone-dissolving compound containing an aromatic moiety. To determine the dissolubility and toxicities of MMP, both in vitro and in vivo experiments were performed using human gallstones and an animal model of gallstones, respectively. Throughout the experiments, MMP demonstrated dissolubility similar to or better than that of MTBE towards both cholesterol and pigmented gallstones, while maintaining lesser toxicities. We believe that the chelate effect of MMP resulted in its improved dissolubility, while its higher boiling point (156 °C) resulted in fewer toxicities. We, thus, believe that MMP could be an attractive alternative to MTBE.

Until now, numerous investigators have developed, or applied, a variety of compounds in dissolving gallstones. The compounds include MTBE, heparin, bile acid, d-limonene, mono-octanoin, EDTA, and sodium hexametaphosphate, all of which are composed of aliphatic chains [15]. Of these, MTBE is the only compound currently available. Although clinical studies have not reported critical toxicities of MTBE to date [4, 24, 25], several studies call into question the safety of MTBE. After administration, MTBE could be absorbed into the duodenum, leading to somnolence, nausea or vomiting [5–9, 11–14], it can also be systemically absorbed, resulting in hemolysis and reversible kidney injury [26]. It was reported that inhalation of MTBE led to a statistically significant increase in kidney tumors and liver tumors in rats [27]. Furthermore, MTBE has resulted in the development of cancers in many organs and tissues, which were similar to those caused by exposure to equal doses of carcinogens such as benzene, vinyl chloride, and 1,3-butadiene [28]. Oral exposure to MTBE has been shown to cause dose-dependent, statistically significant increases in carcinomas such as lymphoma, leukemia, and Leydig cell carcinoma of the testes in rats [29]. These dismal outcomes of MTBE motivated us to attempt to develop a novel gallstone-dissolving compound.

Basically, MMP is an MTBE analog with an aromatic moiety. MTBE is an asymmetric chemical compound with an ether functional group; one side of MTBE has a bulky tert-butyl group and the other side has a simple methyl group. MMP has a structure wherein the bulky aliphatic tert-butyl group of MTBE is replaced with an aromatic functional group while keeping the methyl group intact on the other side. The compounds with an aromatic ring have a relatively higher boiling point and lower vaporized pressure. MMP thus boils at a relatively higher temperature (156 °C) and, thus, evaporates less at room temperature, resulting in lesser toxicities while preserving the dissolubility of MTBE.

Our study suggests that the improved dissolubility of MMP is partly because it acts as a chelating ligand. Chelate is a complex compound consisting of a central metal atom and a large molecule (a ligand) that is attached to the metal atom in a cyclic manner. The chelate effect, thus, refers to the enhanced affinity of chelate ligands for a metal ion compared to the affinity of the non-chelating ligands. The chelate effect promotes gallstone dissolubility, as demonstrated by the enhanced dissolubility of calcium stones by EDTA, the representative chelating agent [21]. MTBE does not have a chelating property because it has only one heteroatom with a lone pair of electrons. In contrast, MMP has two nearby heteroatoms with lone pair electrons, thus enabling to chelate the calcium component in gallstones. Additionally, the aromatic ring in MMP, named pyridine, has the advantage of being miscible with water and virtually all organic solvents, thereby facilitating gallstone dissolution. In the NMR test, we found that when the equivalent of Co(ClO_4_)_2_·6H_2_O was increased to 1.0–2.0 equivalents relative to the solvents, MMP exhibited considerably higher chemical shift than did MTBE, suggesting the enhanced affinity by chelate effect between MMP and Co^2+^. Our in vitro and in vivo studies demonstrated that MMP showed higher dissolubility than MTBE in both models of cholesterol and, more prominently, pigmented gallstones. Therefore, we believe that MMP potentiates the dissolubility of calcium-rich pigmented gallstones by chelating the calcium component, which needs to be validated in further studies.

MTBE or MMP are basically cholesterol solvents; therefore, they exhibit higher dissolubility for cholesterol gallstones than pigmented gallstones. In our in vitro study, whereas MTBE showed 65.7%, 54.2%, and 29.0% 24-h-dissolubility, MMP showed 88.2%, 61.0%, and 50.8% 24-h-dissolubility for cholesterol, mixed, and pigmented gallstones, respectively. The higher dissolubility for cholesterol gallstones is clinically preferable because most gallstones predominantly consist of cholesterol (> 85%) in the developed countries [30, 31].

Currently, topical gallstone-dissolving agents are restrictively available for patients who refuse surgery or are at high risk of surgery, possibly due to the concerns for their serious toxicities as well as the wide recognition of laparoscopic cholecystectomy as a safe operation. In addition, it remains controversial whether the complete removal of gallbladder can be justified in patients with gallstones with a functioning gallbladder, because although it is minimal, surgery inevitably accompanies a certain degree of operative risks and occasionally leads to postcholecystectomy syndrome [32]. Finally, although still controversial, numerous epidemiological investigations and meta-analyses indicate that cholecystectomy can be a risk factor for gastrointestinal cancers [33–37]. The reason is frequently attributed to the deleterious effects of bile acid exposure; it acts like a carcinogen, in terms of enhancing DNA damage, stimulating mutation, inducing apoptosis in the short term, and ultimately promoting apoptosis resistance in the long term [33]. Therefore, we believe that there are still indications for gallstone-dissolving solvents in lieu of laparoscopic cholecystectomy.

Several obstacles are placed in front of the popular use of gallstone dissolution therapy. The first concern is high incidence of stone recurrence. In a study of enrolling 803 patients with gallstones who had received contact litholysis using MTBE, stone recurrence rate was about 40% in solitary stones and about 70% in multiple stones over 5 years [4]. Another challenge is to obtain the minimally invasive way of introducing the solvent into gallbladder. The last, but not least concern is safety because it is almost inevitable to have the proportion of solvent to be absorbed into duodenum or systemic circulation.

This study has several limitations. In the in vivo experiments using hamsters, although there was random assignment of each group, blind assessment of outcomes by investigators was not achieved. In addition, during experiments using hamsters, we could not compare the weights of gallstones before and after treatment because it was nearly impossible to measure the weights of gallstones before the treatment. Instead, in vivo gallstone dissolubility of each solvent was determined by comparing the weights of solvent-treated gallstones and control (DMSO)-treated gallstones.

## Conclusion

In conclusion, MMP was found to have similar or higher dissolubility of gallstones and lesser toxicity than MTBE. Representatively, our in vitro experiments determined MMP to have 1.34 times (88.2% vs. 65.7%) higher 24-h dissolubility for cholesterol gallstones, and 1.75 times (50.7% vs. 29.0%) higher dissolubility for pigmented gallstones. Presently, the wide application of MTBE is limited by several toxicities, most of which originate from its higher evaporation rate due to a relatively lower boiling point. MMP wound be preferable to MTBE because of its lower evaporation rate and similar or higher dissolubility possibly by an additional chelating effect. If clinical trials support these conclusions, contact litholysis by MMP would not only replace conventional MTBE treatment considerably, but it will also be an attractive alternative to laparoscopic cholecystectomy in managing certain patients with gallstones.

## Additional files

**Additional file 1.** Diet composition of each group.

**Additional file 2. ** Additional Materials and Methods.

**Additional file 3. ** H-NMR data of MTBE and MMP.

**Additional file 4. ** Ultraviolet-visible electronic absorbance data and fluorescence spectrometer data of each solvent.

**Additional file 5. ** The change of NMR spectroscopy of MTBE with increasing Co^2+^ cation.

**Additional file 6. ** The change of NMR spectroscopy of MTBE with increasing Ca^2+^ cation.

**Additional file 7. ** The change of NMR spectroscopy of MMP with increasing Co^2+^ cation.

**Additional file 8. ** The change of NMR spectroscopy of MMP with increasing Ca^2+^ cation.

**Additional file 9. **  Weight changes of hamsters in the groups of cholesterol gallstones and of pigmented gallstones, respectively.

**Additional file 10. ** Determination of tissue toxicities of each solvent on 1 day after oral administration.

**Additional file 11. ** Determination of tissue toxicities of each solvent on 14 days after oral administration.

## Data Availability

All data generated or analysed during this study are included in this published article and its additional files. For any additional information, please contact the corresponding author.

## Abbreviations

Bax: bcl-2-like protein 4
CNS: central nervous system
DMSO: dimethyl sulfoxide
EDTA: ethylenediamine tetraacetic acid
ELISA: enzyme linked immunosorbent assay
hGBEC: human gallbladder epithelial cell
IHC: immunohistochemistry
IL-6: interleukin-6
Mcl-1: myeloid cell leukemia 1
MMP: 2-methoxy-6-methylpyridine
MTBE: methyl-tertiary butyl ether
NMR: nuclear magnetic resonance
PCNA: proliferation cell nuclear antigen
TGA: thermogravimetric analysis
TNF-α: tumor necrosis factor-α
