# Supplementary material for: Efficacy and safety of a novel topical agent for gallstone dissolution: 2-methoxy-6-methylpyridine
Source: J Transl Med. 2019 Jun 10;17:195. doi: 10.1186/s12967-019-1943-y (PMC6558798; doi:10.1186/s12967-019-1943-y)
Supplement: Supplementary file 2 — Additional file 2. Additional Materials and Methods. [file 12967_2019_1943_MOESM2_ESM.docx]

**<Additional Experimental Procedure>**

**Ultraviolet (UV) absorption**

To measure the steady-state absorption and emission spectra, we used an UV–vis-NIR spectrophotometer (SCINCO S-4100 Diffuse Reflectance-Ultraviolet/Visible, Seoul, Republic of Korea) equipped with photo diode array (PDA) photomultiplier detector (photo diode array), CW Xenon lamp, and a fluorescence spectrophotometer (Perkin Elmer LS-55 Fluoroscence Spectrometer; Perkin Elmer, Waltham, MA). The phosphorescence spectra were recorded by a photomultiplier tube (PMT) detector equipped to a monochromator (Perkin Elmer). The temporal profiles were measured using a monochromator equipped with a photomulti-plier (CR 131; Zolix Instruments Co., Beijing, China) and a digital oscilloscope (TDS-784D; Tektronix, Salem, Oregon). Oxygen in sample solutions was removed through the argon-pursing method. The sub-picosecond time-resolved absorption spectra were collected by a pump-probe transient absorption spectroscopy system. The pump light was generated by a regenerative amplified titanium sapphire laser system (Spitfire Ace [1 kHz], Santa Clara, CA) pumped by a diode-pumped Q-switched laser (Spectra Physics, Santa Clara, CA). The seed pulse was generated by a titanium sapphire laser (Spectra Physics). The pulses (200 and 800 nm) generated from an optical parametric amplifier were used as the excitation pulse. A white light continuum pulse was generated by focusing the residual of the fundamental light to a thin Sapphire crystal after the controlled optical delay, and the probe beam was detected with the CCD detector installed in the absorption spectroscopy. The pump pulse was chopped by the mechanical chopper synchronized to one-half of the laser repetition rate, resulting in a pair of the spectra with and without the pump, from which absorption change was measured.

**Preparation of human gallbladder epithelial cells**

Human gallbladders were obtained after cholecystectomy performed at the Daejeon St. Mary’s hospital, the Catholic University of Korea. Human gallbladder epithelial cells (hGBECs) were isolated from the gallbladder by trypsin digestion method.^45^ GBECs were maintained in DMEM/F12 medium (Thermo Scientific, Carlsbad, CA). The medium was supplemented with 10% fetal bovine serum (FBS; HyClone, Logan, UT) and 1% penicillin-streptomycin antibiotics (Thermo scientific) at 37°C in a humidified atmosphere with 5% CO_2_ in incubator.

**Western Blotting Analysis**

The cells were washed two times with ice-cold phosphate buffer solution (PBS) and then lysed in EzRIPA Lysis kit (ATTO Corporation, Tokyo, Japan) which includes protease inhibitor and phosphatase inhibitor. After centrifugation at 12,000×g for 10 min, the supernatant was collected, and protein concentration was determined using Bradford reagent (Bio-Rad, Hercules, CA). Samples of equal amount of protein (30 μg) were loaded per well, separated on SDS-polyacrylamide gels, and then electrophoretically transferred onto nitrocellulose membranes. The membranes were then incubated with a blocking buffer (Biofact, Daejeon, Republic of Korea) at 23.5°C for 1 h. Subsequently, the membranes were incubated with primary antibodies (1:1,000 dilution) at 4°C overnight and then incubated with HRP-conjugated secondary antibodies (1:2,000 dilution) for 1 h at 23.5°C. The following antibodies were used: proliferation cell nuclear antigen (PCNA), myeloid cell leukemia 1 (Mcl-1), β-actin, HRP conjugated anti-rabbit IgG, and HRP conjugated anti-mouse IgG (All from Cell Signaling, Beverly, MA). Specific immune complexes were detected using Western Blotting Plus Chemiluminescence Reagent (Millipore, MA).

**Enzyme linked immunosorbent assay (ELISA)**

After obtaining the sera from the hamsters at 24 h following solvent exposure, and the serum interleukin-6 (IL-6) and tumor necrosis factor-α (TNF-α) concentrations were determined using ELISA kits (MyBioSource, San Diego, CA).

**Immunohistochemical analysis**

Paraffin-embedded tissue sections were deparaffinized in xylene and rehydrated in a graded series of alcohol. The antigen was retrieved with 0.01 M citrate buffer (pH 6.0) by heating the sample in microwave oven for 10 min. The tissue sections were then placed in 3% hydrogen peroxide for 3 min to inactivate the endogenous peroxidase, and blocked for 10 min with normal horse serum in Vectastain ABC kit (Vector Laboratories, Burlingame, CA). The primary antibodies used for this study were cleaved caspase-3 and PCNA (All from MyBioSource). The pre-diluted primary antibodies were applied for overnight at 4℃. The slides were then treated with biotinylated secondary antibody for 20 min at 23.5°C, developed by immPACTTM peroxidase substate kit (Vector Laboratories) for 10 min at 23.5°C.
