# Supplementary figures and images for: Efficacy and safety of a novel topical agent for gallstone dissolution: 2-methoxy-6-methylpyridine
Source: J Transl Med. 2019 Jun 10;17:195. doi: 10.1186/s12967-019-1943-y (PMC6558798; doi:10.1186/s12967-019-1943-y)

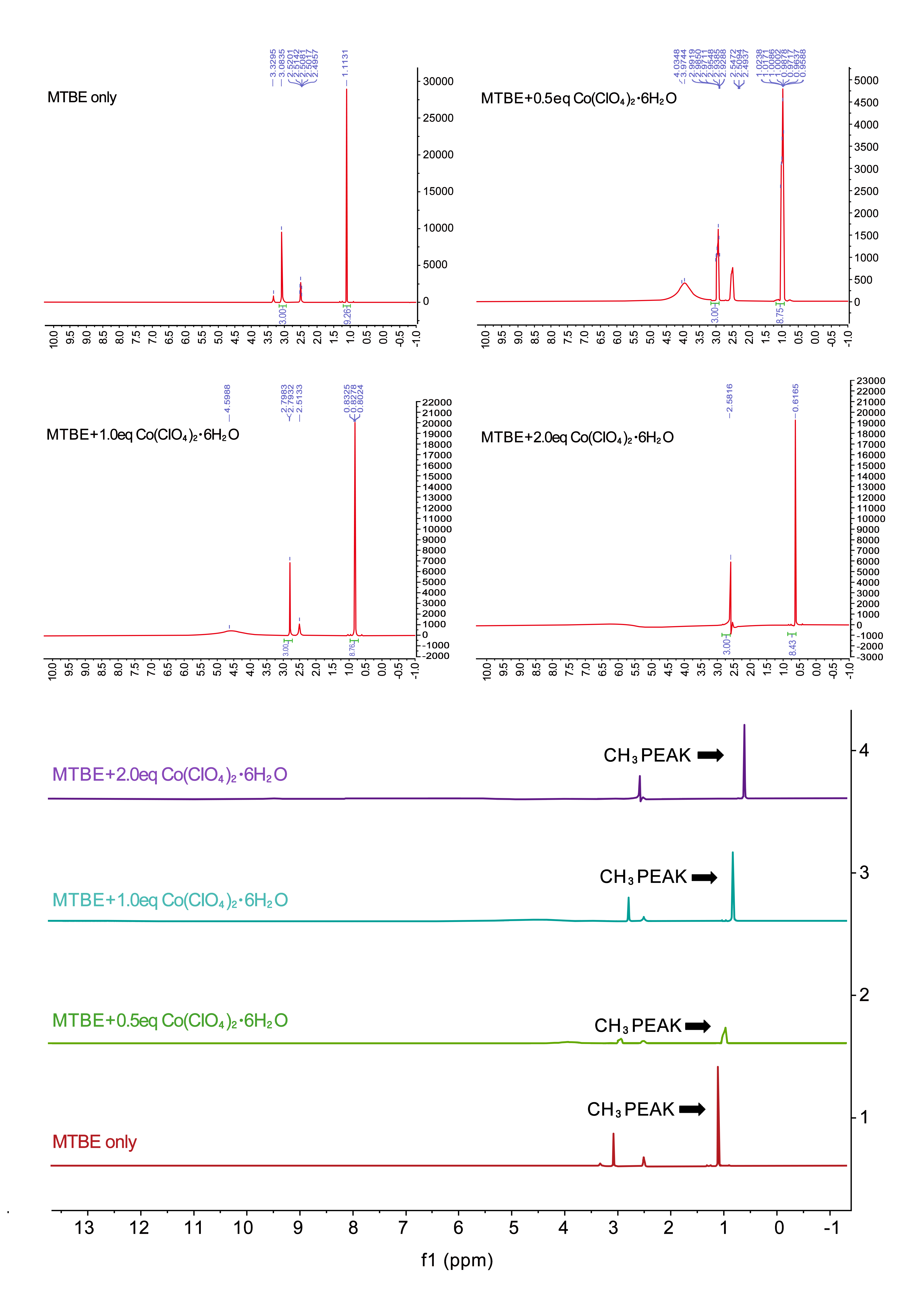

Supplement: Supplementary file 5 — Additional file 5. The change of NMR spectroscopy of MTBE with increasing Co2+ cation. [file 12967_2019_1943_MOESM5_ESM.tif]

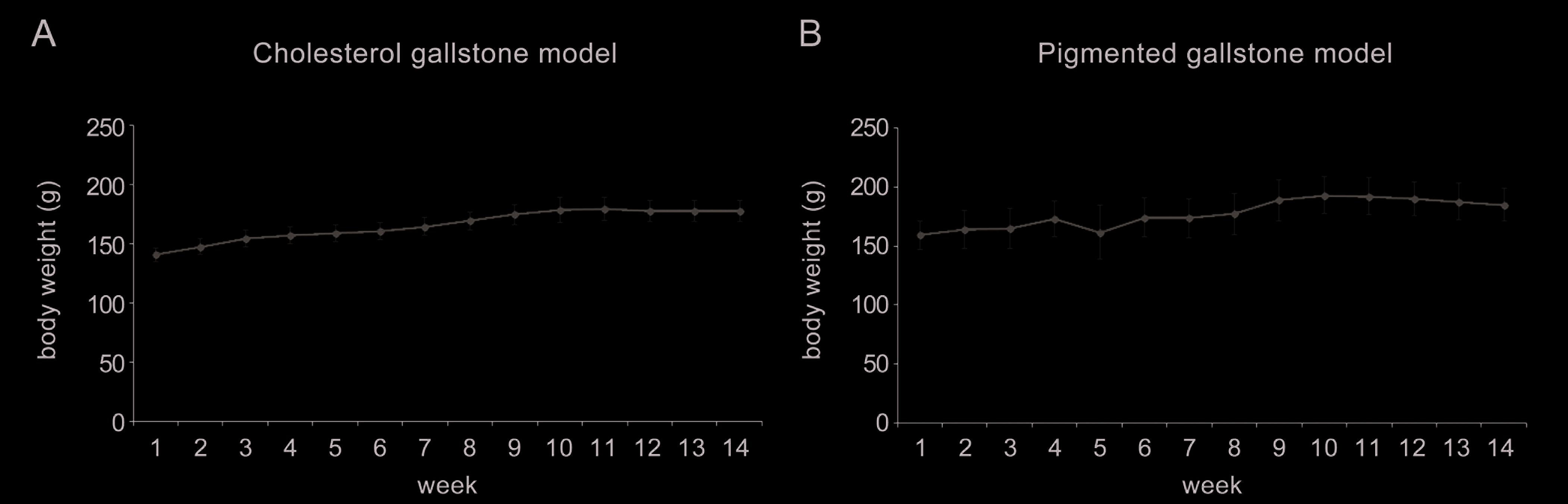

Supplement: Supplementary file 9 — Additional file 9. Weight changes of hamsters in the groups of cholesterol gallstones and of pigmented gallstones, respectively. [file 12967_2019_1943_MOESM9_ESM.tif]
